# Emotional eating and mental health of nurses working in Lebanese hospitals during the double crisis

**DOI:** 10.1017/jns.2024.63

**Published:** 2024-10-09

**Authors:** Rosy Nahed Mitri, Zeina El-Ali, Maha Dankar

**Affiliations:** 1 Department of Nutrition & Dietetics, Faculty of Health Sciences, Beirut Arab University, Tripoli, Lebanon; 2 Department of Nursing, Faculty of Health Sciences, Beirut Arab University, Tripoli, Lebanon

**Keywords:** COVID-19, Eating habits, Emotional eating, Mental health, Nurses

## Abstract

The 2019 coronavirus (COVID-19) pandemic and strict quarantine increased the likelihood of mental symptoms and abnormal eating behaviours. This study aimed to assess the magnitude of emotional eating (EE) among nurses working in Lebanese hospitals and its association with mental health. A cross-sectional study was conducted among nurses aged between 18 and 50 years working in Lebanese hospitals during the COVID-19 outbreak and the economic crisis. A total of 303 nurses consented to participate. The mean EE score was 28.56 (±8.11). The results of this study revealed that 53.8% of the nurses reported depression, 58.1% suffered from anxiety and 95.1% experienced either moderate or severe stress. The study concluded that females (β = 8.112, P = 0.004), non-smokers (β = –4.732, P = 0.01) and depressed nurses (β = 0.596, P = 0.046) had a higher tendency towards EE. Additionally, it was found that EE was associated with weight gain (β = 6.048, P = 0.03) and increased consumption of fried foods (β = 5.223, P = 0.001). Females experienced more stress (β = 2.244, P = 0.003) and anxiety (β = 1.526, P = 0.021) than their male counterparts. With regard to mental health, depression was associated with weight gain (β = 2.402, P = 0.003) and with lower consumption of healthy foods such as nuts (β = –1.706, P = 0.009) and dishes prepared with sofrito sauce (β = –1.378, P = 0.012). These results can help the health authorities to design preparedness plans to ensure proper mental and physical well-being of nurses during any unforeseen emergencies.

## Introduction

The COVID-19 outbreak and the public health policies implemented in reaction to it have had negative physical, psychological and mental health consequences for people from all age groups.^(1)^ In Lebanon, a country already hit by economic crisis and political instability, the effect of the pandemic on mental health has been even more deleterious. Indeed, a study conducted in 2020 revealed that the prevalence of anxiety and depression in Lebanon is about 42% and 42.6% respectively.^(2)^ These numbers are considerably higher than those reported in a sample of Lebanese adults interviewed between September 2002 and 2003, where the prevalence of anxiety disorders was 16.7% and the prevalence of major depression disorders was 9.9%.^(3)^

Concomitantly with the Covid-19 outbreak, Lebanon was undergoing a severe economic crisis, therefore the country was undergoing a so-called “double crisis”. In most countries, even though most restrictions due to the pandemic were lifted by 2022, the stress and the burden on the fragile Lebanese healthcare system wasn’t resolved yet due to the ongoing economic depreciation affecting this system. The devaluation of the Lebanese currency has caused major challenges for many hospitals in Lebanon, making it harder for them to buy essential healthcare supplies.^(4)^

Negative emotions such as worry, irritation or depression can lead to a compulsive need to eat, or so-called emotional eating (EE).^(5)^ EE is characterised by the inability to discern between biological hunger signs and the desire to eat to cope with unpleasant feelings.^(6)^ Eating in response to negative feelings, such as those experienced during the COVID-19 pandemic, can therefore lead to weight gain, which can subsequently adversely affect individuals’ health. In this regard, a Norwegian cohort study revealed that during the COVID-19 pandemic, psychological distress was associated with weight gain, and this association was partially mediated by emotional eating.^(7)^

When it comes to the association between mental health and emotional eating, nurses are of particular concern. Working irregular shifts, such as mornings, evenings and, especially, night shifts, can result in increased calorie consumption, obesity and a poor-quality diet.^(8)^ The distribution of energy and nutrient intake has been found to vary based on work shift, with lipid and protein intake for rotating shift workers being associated with higher consumption of beef, eggs, juices, and pasta.^(9)^ This is not consistent with nutritious eating regimens like the Mediterranean diet (MD) which is one of the healthiest dietary patterns, emphasising fruits, vegetables, grains, nuts, and greens intake with low to moderate consumption of dairy products (yogurt, cheese), and poultry, with limited consumption of sweets, bakery goods and red meat.^(10)^ Additionally, the nursing profession is known as being a stressful job, due to the complexity of the assigned tasks.^(11)^ During the pandemic, the mental and emotional health of nurses was seriously compromised. Nurses experienced greater rates of depression, anxiety and insomnia during this pandemic (37%, 35% and 43%, respectively), higher rates compared to those during other pandemics, such as Middle East respiratory syndrome (MERS) and Severe acute respiratory syndrome (SARS).^(12)^ Therefore, nurses are very likely to succumb to stress, as well as to adopt unhealthy dietary and lifestyle habits. Moreover, previous studies have associated their mental health with eating habits. In this regard, a cross-sectional study in Turkey revealed that perceived stress is positively associated with EE among nurses and the authors concluded that special programmes should be developed to enable nurses to make healthy dietary choices.^(13)^ Therefore, exploring the extent of EE and its association with mental status among nurses during pandemics and emergencies in different contexts is important to develop well-tailored and culture-specific preparedness plans that will allow nurses to cope with any unexpected health emergencies in the future without succumbing to EE that has a tremendous effect on their health and well-being. To the best of our knowledge, no previous studies have examined the burden of EE among nurses working in Lebanese hospitals during the double crisis and its association with mental health.

Given the nature of their job and the deleterious effect of the double crisis on their mental health, we hypothesised that nurses working in Lebanese hospitals are more likely to experience high levels of EE during the pandemic and that this eating behaviour is associated with the burden of the crisis on their mental health. Therefore, the main aim of our study is to assess the magnitude of EE among nurses working in Lebanese hospitals and its association with mental health. Factors correlated with each of EE and mental health, including adherence to MD, will also be investigated to gain a better understanding of the situation as a whole. This study will enable the Lebanese Government to design specific measures in order to keep nurses resilient during any unexpected health emergencies.

## Methodology

### Study design and participants

A cross-sectional study was conducted between March and May 2022 among nurses aged between 18 and 50 years working in Lebanese hospitals during the COVID-19 outbreak. Exclusion criteria included the presence of at least one self-reported non-communicable disease (NCD) (e.g. thyroid disorders, diabetes mellitus, cancer), or being pregnant or lactating. An online questionnaire was developed using Google Forms. A total of 750 nurses working in eleven hospitals (8 private and 3 governmental) were invited to participate via WhatsApp and emails. Only 335 nurses consented to participate in the study (44.6% response rate). Among them, 32 were excluded because they met one of the exclusion criteria or because of incomplete and/or random responses. Our final sample size was 303 participants.

### Data collection

Participants completed the questionnaire between March and May 2022. The general aim and information about the ethics of the study were explained at the beginning. The multicomponent questionnaire included four sections that evaluated the degree of emotional eating, sociodemographic characteristics, mental state, health status and eating habits of the participants. The average time for completion was 15 minutes. The questionnaire was pre-piloted with a group of 20 nurses from various departments, to ensure its clarity. Except for four words, which were rephrased, the findings of the questionnaire’s pilot suggested that the questions were clear.

### Sampling procedure

Sample size was calculated based on the mean emotional eating score of 27.5 in Saudi Arabia, using one mean formula (n = sample size; *z* = 1.96, which corresponds to a 95% confidence level; SD = 16, which corresponds to the standard deviation; *d* = 1.8, corresponding to the precision).^(14)^ A minimum number of 300 nurses was needed to estimate a mean with 95% CI and a precision of 1.8. Through the Order of Nurses in Lebanon official email, an online survey was distributed to nurses working in different Lebanese hospitals from March until May 2022. Additionally, nursing directors in a variety of hospitals were contacted to obtain their approval, and the questionnaire was communicated through WhatsApp and email to working nurses.

### Variables

#### Sociodemographic characteristics

These variables included age, gender (male, female), monthly income (enough, not enough), professional title (nurse, registered nurse, head of department, supervisor, director of nursing), shift pattern (always day, always night, day & night), working department (Covid-19, Non-Covid-19), marital status (married, single, divorced), years of experience (<1 year, 1–5 years, 6–10 years, 11–15 years, >15 years), and educational status (TS2/TB3, registered nurse, Master).

#### Emotional eating scale (EES)

The EES comprises 25 self-reported questions evaluating the desire to eat while experiencing negative emotions, such as anger, anxiety or a low mood state (depression). Participants rated their responses on a five-point Likert scale, ranging from 0 (no desire to eat) to 4 (extreme desire to eat). The final score was obtained by adding all of the item scores together, and these varied from 0 to 100, with higher scores reflecting a higher reliance on food in the management of emotions.^(15)^ The Arabic version of the EES was used in this study; it has demonstrated good test–retest reliability (*r* = 0.79, P < 0.001) and internal consistency of 0.81.^(16)^

#### Depressive symptoms (PHQ-9)

The validated Arabic version of the Patient Health Questionnaire-9 (PHQ-9) was used in order to evaluate symptoms of depression experienced by the nurses during the COVID-19 pandemic (Cronbach’s alpha of 0.857).^(17,18)^ On a four-point Likert scale ranging from 0 (never) to 3 (nearly every day), nine questions assessed the incidence of depression symptoms in the previous week. The final scores ranged from 0 to 27, with values of 10 and higher reflecting the existence of depression.^(18,19)^

#### Generalised anxiety disorder (GAD-7)

The GAD-7 has a sensitivity value of 0.83 and a specificity value of 0.84 for identifying generalised anxiety disorder.^(20)^ The anxiety level experienced by the nurses during the previous two weeks was assessed using the validated Arabic version of the GAD-7 which possesses an internal consistency reliability of 0.763.^(17,18)^ Symptoms of anxiety were evaluated on a four-point Likert scale, with responses varying from 0 (never) to 3 (almost every day). The total score fluctuated from 0 to 21, with scores above 10 confirming the existence of generalised anxiety disorder.^(21)^

#### Perceived stress scale (PSS-10)

The Arabic version of the Perceived Stress Scale (PSS) questionnaire was used to quantify the level of stress experienced by the nurses. The scale is composed of ten items, with each question answered on a five-point Likert scale and responses ranging from 0 (almost never) to 4 (very often), with the test-retest reliability having an intra-correlation coefficient of 0.90.^(22)^ Higher values reflected higher levels of perceived stress. The total score was divided into three categories: ≥ 27 = high stress; 14–26 = moderate stress; ≤ 13 = low stress.^(23)^

#### Health and eating habits

Weight and height were self-reported by each nurse in order to calculate their body mass index (BMI) using the following formula: weight (kg)/height (m^2^). BMI was then categorised based on the World Health Organization (WHO) recommendations into four groups: underweight (< 18.5 kg/m^2^), normal weight (18.5–24.9 kg/m^2^), overweight (25.0–29.9 kg/m^2^) and obese (≥ 30 kg/m^2^).^(24)^ Each nurse was also asked to report any weight gain. Furthermore, participants described any change in their physical activity level during the COVID-19 outbreak in the past year.

Regarding eating habits, the Mediterranean Diet Adherence Screener (MEDAS) scale was used in order to evaluate the nurses’ adherence to the MD. The MEDAS is a 14-item questionnaire that evaluates food intake frequencies of certain food items, along with food habits related to the MD. The participants were categorised as low adherent (≤ 5), medium adherent (6–8) and high adherent (≥ 9) to the MD pattern.^(25)^ In addition, each nurse was requested to report whether their intake for each of the above-mentioned 14 items was higher, lower or remained the same during the COVID-19 outbreak. Finally, nurses were asked to describe any change in their snacks, fried food, alcohol, or coffee intakes. Smoking status was also investigated (non-smoker, moderate smoker, heavy smoker).

### Ethical considerations

The study followed the Declaration of Helsinki guidelines and was approved by Beirut Arab University’s Institutional Review Board (date: 20/1/2021, Nb: 2022-H-0136-HS-R-0477). Written approval was obtained from hospital executives. Since the study was web-based, and in order to obtain written consent, the online questionnaire included the sentence: ‘I agree to participate in this study’. Participants were also informed that participation was voluntary and that anonymity and confidentiality would be protected.

### Statistical analysis

The data was analysed using SPSS software version 22, using mean and standard deviations for continuous variables, and frequencies and percentages for categorical variables. Pearson’s correlation was used for linear correlation between continuous variables. A bivariate analysis was conducted to examine factors associated with EE, depression, anxiety and stress, using independent sample *t*-tests for two means and ANOVA tests for three or more means. Multiple regression analysis was performed for variables significantly correlated with EE, depression, anxiety and stress, and for the correlation between EE and mental health, with a P-value < 0.05 indicating statistical significance.

## Results

The baseline characteristics of the study sample are represented in Table 1. The mean age of the participants was 28.56 ± 8.11 years, and the average EE score was 37.9 (± 10.1). More than two-thirds of the nurses (73.9%) reported that their income was insufficient. Unhealthy weight status was evident among the nurses, with 40.9% of them reporting an increase in their weight during the pandemic. Additionally, 44.2% of the nurses were overweight or obese. This fact coincided with a low level of physical activity, with 39.6% reporting a decrease in their level of physical activity. In addition, the nurses reported a poorer-quality diet, with only 11.9% describing good adherence to the MD.

**Table 1.** General characteristics of the study population (n = 303)

Variables	Frequency	Percentage
Age		
Mean (±SD)	28.56 (8.11)	
Emotional eating (EE)		
Mean (±SD)	37.9 (10.1)	
Gender		
Male	80	26.4
Female	223	73.6
Monthly income		
Enough	79	26.1
Not enough	224	73.9
Professional title		
Nurse	175	57.8
Registered nurse	107	35.3
Head of department	13	4.3
Supervisor	5	1.7
Director of nursing	3	1
Work shift		
Always day	139	45.9
Always night	39	12.9
Day & night	125	41.3
Department		
Covid-19	53	17.5
Non covid-19	250	82.5
Experience in years		
<1	80	26.4
1–5	104	34.3
6–10	50	16.5
11–15	27	8.9
>15	42	13.9
Marital status		
Married	105	34.7
Single	185	61.1
Divorced	13	4.2
Educational status		
TS2/BT3	105	34.7
Registered nurse (LA-LT-BS)	148	48.8
Master	50	16.5
Smoking		
No smoking	192	63.4
Moderate smoking	85	28.1
Heavy smoking	26	8.6
Weight gain		
Yes	124	40.9
No	179	59.1
Body mass index (BMI)		
Underweight	7	2.3
Normal weight	162	53.5
Overweight	84	27.7
Obese	50	16.5
Consumption of 3 regular meals		
Never	69	22.8
Sometimes	181	59.7
Most of the time	20	6.6
Every day	33	10.9
Fried food consumption		
More than usual	78	25.7
Less than usual	155	51.2
No change	70	23.1
Physical activity		
Increase	43	14.2
Decrease	120	39.6
No change	64	21.1
I don’t usually practice	76	25.1
Alcohol drinking		
More than usual	2	0.7
Less than usual	11	3.6
No change	14	4.6
Caffeinated beverages intake		
More than usual	132	43.6
Less than usual	38	12.5
No change	96	31.7
Usually No	37	12.2
Med diet adherence		
Low adherence	111	6.6
Moderate adherence	156	51.5
High adherence	36	11.9

TS: Technique Superieur, BT: Baccalaureat Technique, LT: License Technique, BS: Bachelor of Science.

EE: Emotional Eating.

**Fig. 1.**
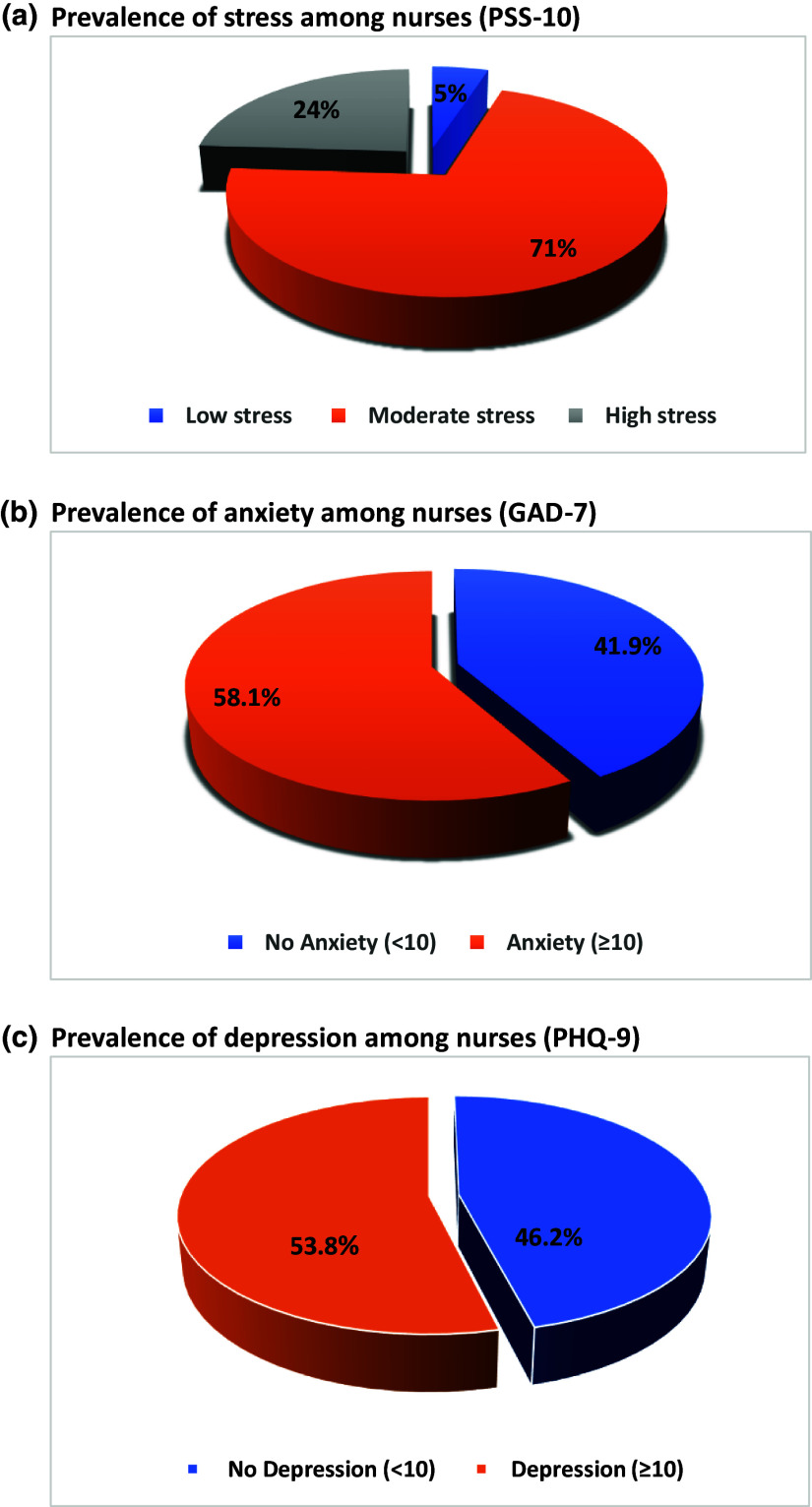
Mental state of the nurses. (a) Prevalence of stress among nurses (Perceived Stress Scale-10). (b) Prevalence of anxiety among nurses (Generalised anxiety disorder-7). (c) Prevalence of depression among nurses (Patient Health Questionnaire-9).

Figure 1 represents the mental state of the participants. A poor mental state was evident among the majority of the nurses, with 53.8% reporting depression, 58.1% suffering from anxiety and 95.1% experiencing either moderate or severe stress.

Table 2 displays the factors associated with emotional eating and the mental state of the participants during the COVID-19 pandemic, through a bivariate analysis. With regard to EE, female nurses manifested a higher EE than males (42.46 ± 7.96, 36.4 ± 6.62, respectively; P = 0.038). Similarly, those with insufficient income reported greater EE (42.97 ± 7.61) than nurses with sufficient income (36.22 ± 5.1) (P = 0.016). An advanced academic degree was associated with higher levels of EE, where registered nurses (41.24 ± 8.54) or master degree holders (39.06 ± 7.96) expressed a greater EE compared to those with a technical degree (32.88 ± 6.55) (P = 0.006). In addition, gaining weight during the pandemic (46.76 ±7.91), and eating more fried food in particular (49.01 ±13.26) (P < 0.001), tended to be associated with higher levels of EE. Excessive intake of caffeinated beverages (43.02 ± 11.37) (P = 0.002) and lower exercise levels (43.95 ± 12.26) (P = 0.003) were also linked to higher EE. However, being a non-smoker (39.75 ± 8.14) was correlated with a higher EE score compared to moderate (38.55 ± 9.90) or heavy smoking (27.96 ± 5.98) (P = 0.032). With regard to stress, female nurses reported a higher stress level (23.27 ± 4.26) than male (21.10 ± 3.59) (P = 0.002). Similarly, anxiety was more prevalent among female nurses than males (11.83 ± 3.62, 10.09 ± 3.01; respectively; P = 0.007), those in a higher position than those in a lower position (16.66 ± 4.14, 10.97 ± 2.58, P = 0.027; respectively), and those reporting a decrease in their physical activity (PA) compared those who increased their PA (22.70 ± 6.15, 10.12 ± 3.42, respectively; P = 0.015). Finally, depression was more prevalent among nurses with insufficient income than those with sufficient income (11.73 ± 3.59, 9.89 ± 2.13, P = 0.032; respectively), those in a higher position compared to those in a lower position (16.67 ± 4.77, 10.90 ± 2.5, P = 0.002; respectively) and those gaining weight compared to those who didn’t gain weight (12.23 ± 2.56, 10.57 ± 2.12, P = 0.030; respectively) during the outbreak.

**Table 2.** Factors associated with EE and mental state among the participants

Variables	Total EE score (Mean ± SD)	P-value	PSS-10 (Mean ± SD)	P-value	GAD-7 (Mean ± SD)	P-value	PHQ-9 (Mean ± SD)	P-value
Age								
	28.56 (8.11)	0.196	28.5 (8.11)	0.951	11.37 (4.95)	0.224	11.24 (4.30)	0.908
Gender								
Male	36.4 (6.62)	0.038	21.10 (3.59)	0.002	10.09 (3.01)	0.007	10.44 (1.56)	0.207
Female	42.46 (7.96)		23.27 (4.26)		11.83 (3.62)		11.53 (2.81)	
Monthly income								
Enough	36.22 (5.1)	0.016	22.41 (5.81)	0.598	10.81 (2.09)	0.0239	9.89 (2.13)	0.032
Not enough	42.97 (7.61)		22.81 (5.92)		11.57 (3.42)		11.73 (3.59)	
Educational status								
TS2/BT3	32.88 (6.55)	0.006	22.48 (6.11)	0.663	11.22 (3.24)	0.658	10.79 (2.61)	0.422
Registered nurse (LA,LT, BS)	41.24 (8.54)		23.01 (3.31)		11.63 (3.75)		11.76 (3.39)	
Master	39.06 (7.96)		22.28 (5.97)		10.96 (3.05)		10.70 (2.98)	
Professional title								
Nurse	37.3 (5.29)	0.067	22.83 (4.32)	0.236	10.97 (2.58)	0.027	10.90 (2.35)	0.002
Registered nurse	40.91 (6.09)		22.91 (4.87)		12.19 (3.72)		12.46 (3.49)	
Head of department	33.38 (7.19)		20.69 (4.06)		10.15 (2.31)		7.46 (1.43)	
Supervisor	22.60 (7.15)		17.80 (3.67)		7.80 (2.02)		4.20 (1.27)	
Director of nursing	17.33 (7.86)		24.67 (5.09)		16.66 (4.14)		16.67 (4.77)	
Work shift								
Always day	33.66 (5.21)	0.604	22.65 (4.52)	0.608	11.13 (2.26)	0.360	11.80 (1.78)	0.139
Always night	35.43 (4.66)		23.56 (5.89)		12.41 (2.76)		13.17 (2.56)	
Day and night	39.13 (7.84)		22.50 (4.89)		11.33 (2.61)		10.83 (2.13)	
Department								
Non Covid-19	37.06 (7.80)	0.143	22.91 (5.83)	0.178	11.42 (3.52)	0.739	11.27 (2.83)	0.889
Covid-19	42.32 (9.16)		21.71 (6.08)		11.16 (3.31)		11.13 (2.42)	
Experiences in years								
<1year	38.23 (6.18)	0.150	22.25 (5.76)	0.698	11.31 (2.61)	0.815	11.05 (3.52)	0.438
1–5 years	40.96 (8.21)		23.30 (6.73)		11.12 (2.38)		11.37 (3.84)	
6–10 years	38.40 (7.03)		22.86 (6.15)		11.14 (2.58)		11.24 (3.65)	
11–15 years	31.00 (5.90)		22.63 (5.96)		12.22 (3.81)		13.18 (4.51)	
>15 years	34.11 (5.81)		21.98 (5.15)		11.86 (3.02)		10.07 (3.21)	
Marital status								
Married	37.58 (5.45)	0.860	22.08 (4.56)	0.114	11.13 (1.69)	0.069	10.57 (2.47)	0.073
Single	38.38 (6.04)		22.86 (5.47)		11.30 (1.71)		11.37 (2.75)	
Divorced	35.46 (8.32)		25.54 (6.82)		14.46 (3.35)		14.92 (3.44)	
Smoking								
No	39.75 (8.14)	0.032	22.63 (6.11)	0.935	11.21 (2.53)	0.507	11.14 (1.94)	0.771
Moderate	38.55 (9.90)		22.75 (6.67)		11.90 (2.64)		11.65 (2.12)	
Heavy	27.96 (5.98)		23.07 (7.24)		10.92 (2.26)		10.73 (2.67)	
Weight gain								
Yes	46.76 (7.91)	<0.001	22.31 (5.41)	0.157	11.12 (1.82)	0.276	10.57 (2.12)	0.030
No	31.89 (5.40)		23.2 (5.23)		11.75 (2.23)		12.23 (2.56)	
BMI								
Underweight	27.00 (5.14)	0.341	22.57 (4.69)	0.158	14.71 (3.98)	0.251	12.88 (3.11)	0.680
Normal	37.54 (7.23)		23.39 (5.86)		11.12 (2.95)		10.845 (2.25)	
Overweight	37.76 (4.13)		22.11 (5.99)		11.30 (2.65)		11.64 (2.32)	
Obese	41.32 (7.91)		21.50 (5.94)		11.86 (5.84)		11.66 (2.65)	
Frequency of eating more								
Yes	43.30 (8.16)	<0.001	22.71 (3.11)	0.985	11.11 (2.31)	0.295	10.91 (2.55)	0.317
No	31.18 (4.94)		22.69 (3.24)		11.73 (2.56)		11.67 (2.31)	
Fried food consumption								
More than usual	49.01 (13.26)	<0.001	22.72 (3.12)	0.318	11.12 (3.18)	0.354	10.91 (2.34)	0.054
Less than usual	34.15 (10.83)		22.69 (3.05)		11.71 (3.25)		11.68 (2.82)	
No change	34.19 (10.05)		22.71 (3.51)		11.11 (3.61)		10.91 (2.56)	
Physical activity								
Increase	41.11 (11.61)	0.003	21.16 (4.12)	0.124	10.12 (3.42)	0.015	10.21 (2.22)	0.157
Decrease	43.95 (12.26)		23.34 (5.45)		22.70 (6.15)		12.00 (3.45)	
No change	34.79 (10.54)		22.01 (5.65)		11.59 (3.95)		10.02 (2.61)	
I don’t usually practice	32.34 (9.86)		23.17 (5.25)		10.36 (3.66)		11.68 (3.17)	
Alcohol drinking								
More than usual	59 (8.11)	0.554	26.50 (5.22)	0.455	12.50 (2.54)	0.393	11.50 (1.90)	0.179
Less than usual	38.63 (6.76)		22.90 (4.87)		11.27 (2.86)		14.09 (3.14)	
No change	37.5 (6.88)		24.71 (5.21)		13.57 (3.51)		14.00 (3.24)	
Usually no	37.82 (5.36)		22.57 (5.02)		11.26 (2.62)		10.99 (2.41)	
Caffeinated beverages intake								
More than usual	43.02 (11.37)	0.002	22.43 (4.56)	0.186	11.54 (2.74)	0.501	11.34 (2.95)	0.747
Less than usual	35.00 (9.48)		21.24 (4.23)		10.37 (2.69)		10.16 (2.20)	
No change	33.21 (7.82)		23.21 (5.33)		11.70 (2.56)		11.42 (2.56)	
Usually no	35.43 (8.19)		23.86 (5.47)		10.97 (2.47)		11.59 (2.64)	

TS: Technique Superieur, BT: Baccalaureat Technique, LT: License Technique, BS: Bachelor of Science, EE: Emotional Eating.

PHQ-9: Patient Health Questionnaire-9; GAD-7: Generalised Anxiety Disorder-7; PSS-10: Perceived Stress Scale-10.

P < 0·05 is considered significant.

Table 3 represents the association between the EE and mental health of the participants and the MD. It was apparent that nurses with higher EE consumed more olive oil (P = 0.001) and red meat (P = 0.002), as well as more fats, soft drinks, wine, sweet bakery products, cookies, nuts (P < 0.001) and dishes prepared with sofrito sauce (P = 0.048), compared to other components of the MD. Regarding mental health, a higher stress level was associated with more intake of fats (P = 0.002). In parallel, higher anxiety was correlated with more wine consumption (P = 0.001), but with a lower intake of nuts (P = 0.014) and dishes prepared with sofrito sauce (P = 0.046). Moreover, depression was associated with lower consumption of fruit (P = 0.017), nuts (P < 0.001) and dishes cooked with sofrito sauce (P = 0.015). On the other hand, depressed nurses consumed more red meat (P = 0.017), fats (P = 0.001), soft drinks (P = 0.009) and wine (P = 0.001).

**Table 3.** Factors associated with emotional eating and mental state: the role of the Mediterranean diet

Variables	Total EE score (Mean ± SD)	P-value	PSS-10 (Mean ± SD)	P-value	GAD-7 (Mean ± SD)	P-value	PHQ-9 (Mean ± SD)	P-value
Adherence to MD (total score)								
Low	37.31 (9.86)	0.214	23.46 (6.88)	0.127	11.89 (2.49)	0.144	12.08 (3.05)	0.191
Moderate	37.14 (8.04)		22.50 (5.34)		11.32 (2.12)		10.93 (2.11)	
High	43.66 (11.25)		21.28(4.42)		10.03 (2.05)		10.05 (2.58)	
Olive oil								
Higher	45.95 (9.25)	0.001	23.17 (5.10)	0.460	11.04 (2.19)	0.457	11.51 (3.75)	0.498
Lower	39.62 (7.47)		23.40 (6.88)		12.20 (3.53)		12.17 (2.45)	
As before	35.08 (8.31)		22.39 (5.87)		11.29 (2.04)		10.94 (2.63)	
Vegetables								
Higher	38.98 (6.70)	0.681	21.85 (5.85)	0.141	11.35 (2.74)	0.778	10.90 (2.37)	0.095
Lower	39.52 (7.56)		23.93 (5.31)		11.77 (2.07)		12.91 (2.74)	
As before	37.14 (7.72)		22.59 (6.03)		11.25 (3.01)		10.82 (2.56)	
Fruits								
Higher	40.89 (9.54)	0.224	22.18 (5.48)	0.080	11.41 (2.23)	0.485	11.03 (2.00)	0.017
Lower	39.63 (8.67)		24.10 (6.22)		11.97 (1.97)		13.20 (3.68)	
As Before	36.18 (6.86)		22.33 (5.84)		11.11 (1.00)		10.53 (2.26)	
Red meat								
Higher	50.03 (12.29)	0.002	23.25 (6.28)	0.496	12.46 (2.73)	0.233	12.78 (3.13)	0.017
Lower	36.49 (10.76)		23.03 (5.90)		11.59 (3.17)		12.11 (3.81)	
As before	36.62 (10.60)		22.28 (5.78)		10.93 (2.78)		10.12 (2.09)	
Fats								
Higher	52.87 (13.88)	<0.001	25.84 (5.19)	0.002	12.21 (2.30)	0.278	14.40 (3.98)	0.001
Lower	32.59 (7.86)		22.95 (6.10)		11.75 (2.27)		12.03 (3.90)	
As before	38.48 (10.24)		21.95 (5.68)		10.98 (1.84)		10.14 (2.26)	
Soft drinks or carbonated beverages								
Higher	48.31 (12.16)	<0.001	23.82 (5.84)	0.257	12.27 (4.71)	0.175	13.10 (2.87)	0.009
Lower	32.09 (7.55)		22.60 (6.11)		11.58 (5.28)		11.80 (2.08)	
As before	37.75 (9.45)		22.33 (5.73)		10.89 (4.80)		10.16 (2.97)	
Wine								
Higher	51.20 (14.09)	<0.001	25.30 (7.28)	0.140	15.10 (3.90)	0.001	16.60 (4.13)	0.001
Lower	30.10 (9.69)		23.48 (5.87)		12.66 (3.20)		12.80 (3.68)	
As before	39.77 (12.22)		22.35 (5.79)		10.82 (2.76)		10.53 (3.37)	
Pulses								
Higher	39.01 (11.79)	0.055	23.09 (6.14)	0.596	11.65 (1.10)	0.743	12.26 (3.19)	0.115
Lower	31.92 (9.60)		23.12 (5.90)		11.60 (1.66)		12.03 (3.48)	
As before	39.45 (11.12)		22.41 (5.78)		11.18 (1.00)		10.57 (2.73)	
Fish								
Higher	47.76 (12.42)	0.076	23.82 (6.73)	0.303	12.00 (3.89)	0.159	11.64 (4.35)	0.063
Lower	36.05 (8.43)		23.11 (6.10)		11.89 (3.15)		12.16 (4.83)	
As before	38.65 (9.13)		22.19 (5.55)		10.81(3.11)		10.34 (4.17)	
Sweet bakery and cookies								
Higher	44.04 (11.15)	<0.001	23.78 (6.02)	0.068	12.08 (3.80)	0.051	12.04 (4.04)	0.072
Lower	31.73 (5.73)		22.39 (6.00)		11.74 (3.72)		11.84 (4.60)	
As before	36.91 (8.80)		22.00 (5.60)		10.55 (3.14)		10.20 (3.91)	
Nuts								
Higher	51.50 (12.26)	<0.001	23.40 (6.20)	0.275	11.61 (3.74)	0.014	11.20 (2.86)	<0.001
Lower	32.75 (7.73)		23.12 (6.38)		12.41 (3.45)		13.20 (3.34)	
As before	36.54 (9.27)		22.15 (5.36)		10.56 (3.54)		9.89 (2.96)	
Dishes with sofrito sauce								
Higher	31.08 (5.46)	0.048	22.28 (6.02)	0.059	11.04 (2.78)	0.046	10.49 (2.91)	0.015
Lower	32.52 (8.49)		24.39 (6.24)		12.86 (3.05)		13.52 (2.04)	
As before	38.60 (7.28)		22.35 (5.58)		11.04 (2.96)		10.88 (2.67)	

MD: Mediterranean diet.

PHQ-9: Patient Health Questionnaire-9; GAD-7: Generalised Anxiety Disorder-7; PSS-10: Perceived Stress Scale-10, EE: Emotional Eating.

P < 0·05 is considered significant.

Variables significantly associated with EE or mental health in the bivariate analysis were included in a multiple linear regression analysis (Table 4). Female gender (β = 8.112, P = 0.004), smoking (β = –4.732, P = 0.010), weight gain (β = 6.048, P = 0.03), eating more (β = 0.461, P = 0.032) and consuming more fried items (β = 5.223, P = 0.001) were significantly associated with EE in this regression analysis. With regard to stress and anxiety, only the female gender was significantly associated with higher stress (β = 2.244, P = 0.003) and anxiety levels (β = 1.526, P = 0.021). Furthermore, higher levels of depression were correlated with weight gain (β = 2.402, P = 0.003) and with a reduced consumption of nuts (β = –1.706, P = 0.009) and dishes cooked with sofrito sauce (β = –1.378, P = 0.012) (Table 4). Finally, among the studied mental health problems, a positive association was detected only between depression and EE (β = 0.596, P = 0.046) (Table 5).

**Table 4.** Factors associated with emotional eating and mental health among nurses: Multiple regression analysis

	Total EE		PSS-10		GAD-7		PHQ-9	
	Beta	P-value	Beta	P-value	Beta	P-value	Beta	P-value
Gender	8.112	0.004	2.244	0.003	1.526	0.021		
Monthly income								
Educational status								
Smoking	–4.732	0.010						
Weight gain	6.048	0.030					2.402	0.003
Eating more during COVID-19	0.461	0.032						
Fried food intake	5.223	0.001						
Physical activity								
Caffeinated beverages intake								
Olive oil intake								
Red meat								
Fats								
Soft drinks								
Wine								
Sweet bakery & cookies								
Nuts							–1.706	0.009
Dishes with sofrito sauce							–1.378	0.012
Adjusted R^2^	0.247		0.026		0.032		0.066	
F	6.842		5.035		2.657		3.139	
P-value	<0.001		0.007		0.016		0.001	

PHQ-9: Patient Health Questionnaire-9; GAD-7: Generalised Anxiety Disorder-7; PSS-10: Perceived Stress Scale-10; EE: Emotional Eating *P < 0.05 statistically significant.*

**Table 5.** Association between EE and mental health of the participants

	Total EE	
	Beta	P-value
PSS-10	–0.140	0.584
GAD-7	–0.540	0.187
PHQ-9	0.596	0.046
Adjusted R^2^	0.003	
F	0.349	
P-value	0.0258	

PHQ-9: Patient Health Questionnaire-9; GAD-7: Generalised Anxiety Disorder-7; PSS-10: Perceived Stress Scale-10; EE: Emotional Eating.

P < 0.05 is statistically significant.

## Discussion

To the best of our knowledge, this is the first study to assess the magnitude of EE among nurses working in Lebanese hospitals during the COVID-19 outbreak and the economic crisis and its association with mental health. Our results showed that during the double crisis witnessed by the Lebanese healthcare system, the mean EE score was 28.56 (±8.11) and the prevalence of mental symptoms was high with 53.8% of the nurses suffering from depression, 58.1% from anxiety, and 95.1% from stress. In addition, the study concluded that depressed nurses, females, non-smokers, those who gained weight, and those who ate more, and in particular those who consumed more fried foods, had a higher tendency towards EE. Regarding mental health, gender difference was noted for the tendency towards stress and anxiety, with females experiencing more stress and anxiety than their male counterparts. Depression was associated with weight gain and with lower consumption of healthy food items (dishes prepared with sofrito sauce and nuts).

Previous studies conducted in various countries found high levels of EE among the healthcare staff during the COVID-19 outbreak. For instance, in Qatar, nurses working during the COVID-19 outbreak had increased odds of experiencing EE, with levels 2.62 times higher than during the pre-pandemic period.^(26)^ In our study the mean EE score in our sample was 37.9 (±10.1) however, due to the use of different tools to assess EE among nurses, comparisons of previous findings to our results were impossible. However, a survey conducted on young Saudi Arabian women during the epidemic revealed a mean EE score of 27.5.^(14)^ These findings spotlight the magnitude of EE among nurses working in Lebanese hospitals during that period.

Our results showed that 43.3% of the nurses were eating more during the double crisis and this increased food consumption was associated with EE. Furthermore, EE was positively associated with weight gain in our sample of nurses. The relationship between EE and weight gain has been extensively highlighted in previous studies. In a two-year cohort study, higher emotional eating among employees predicted more weight gain, independently of other factors such as smoking, alcohol and other dietary habits.^(27)^ Moreover, emotional eaters struggle with losing weight. They are half as likely as non-emotional eaters to accomplish the 10% weight loss goal of standard behavioural weight loss intervention programmes.^(28)^ In addition to excessive food intake, the dietary choices of the emotional eaters could also contribute to weight gain. Emotional eaters usually seek comfort food, and previous research theorised that, during the enforced quarantine, EE arose as a coping mechanism to reduce uncomfortable feelings.^(29)^ In fact, the COVID-19 outbreak impacted people’s eating habits in general, with many reporting engaging in harmful eating behaviours, such as reaching for comfort food that is poor in nutrients and high in calories.^(30)^ In China, even among healthcare workers who had a healthy diet prior to COVID-19, a decreased consumption of vegetables (P = 0.027) and an increased intake of soft drinks (P = 0.003) and convenience food (P < 0.001) were reported during the outbreak. Our results showed that among other food choices, higher EE scores were associated with consuming more fried foods in particular; therefore, it could be that fried items are perceived as being comfort food by our population. In fact, serotonin and dopamine, which have been shown to be positive emotion enhancers, are released more readily when fats and foods high in refined carbohydrates are consumed.^(31)^

Gender emerged as another factor associated with EE, with female nurses reporting more emotional eating than males. This is consistent with previous studies, where female nursing students have experienced significantly higher levels of emotional eating compared to their male counterparts.^(32)^ Similarly, in Turkey, female emergency service workers had higher EE scores than males (P = 0.022).^(33)^ This result can be attributed to gender disparity in food intake, where women generally show less dietary control than men. Women are more inclined than males to report eating compulsively and out of control.^(34)^ For example, the average lifelong prevalence of developing a binge eating disorder is 3.5% for females in comparison to 2.0% in males.^(35)^

In our study, heavy smokers had a lower EE score compared to non-smokers and those who smoke in moderation (P = 0.018). This could be explained by the fact that when faced with an unusual or unexpected situation, people tend to choose a coping mechanism, which could be either food—favouring empty-calorie items—or smoking, to lessen the burden of their negative feelings. There is compelling evidence that nicotine reduces feelings of tension and rage.^(36)^ Smokers claim that they smoke more frequently when they are upset or angry because they believe that smoking will make them feel better.^(37)^

Our results shed the light on the extent of mental symptoms among nurses working in Lebanese hospitals during COVID-19 outbreak and the economic crisis. More than half of the nurses were suffering from depression or anxiety (53.8% and 58.1% respectively) and even worse, the big majority were experiencing moderate or severe stress (95.1%). This rate is dramatically higher than the one reported before 2019, where the prevalence of depression was only 36.2% among Lebanese nurses.^(38)^ Mental symptoms were previously reported among nurses during the COVID-19 outbreak, i.e. a study conducted in Iran during the COVID-19 outbreak, found moderate levels of stress, depression and anxiety among a group of nurses working in an educational hospital.^(39)^ However, our findings revealed higher rates of mental symptoms than the ones found in many other countries, in fact a systematic review and meta-analysis revealed an overall prevalence of stress of 43%, while anxiety and depression were 37% and 35%, respectively.^(12)^ Although the toll of COVID-19 cases in Lebanon was significantly lower than the one in China, our rates of mental symptoms were comparable to those found in Wuhan, China, where depression and anxiety symptoms were present in 58% and 54.2% of healthcare workers respectively.^(40)^ This finding is alarming, it pinpoints the fact that the pandemic had a higher-than-expected impact on nurses in Lebanon. At first sight, it may seem that the nurses in Lebanon had an exaggerated response to the emergency situation, however taking into account the economic situation in the country and the realities of the healthcare system at that time, one can understand the magnitude of the mental burden these professionals were experiencing. While the world was facing the COVID-19 pandemic, Lebanon was dealing with a double crisis – the pandemic as well as an economic crisis—which has led to the worst rates of unemployment, inflation and poverty that Lebanon has ever experienced.^(41,42)^ Due to the pandemic, Lebanese nurses were forced to take double shifts to replace sick or rotated nurses, in addition to facing medication shortages and high daily death rates.^(43)^ Simultaneously, they were underpaid due to the economic crisis, which led to a remarkable decline in the Lebanese pound that has lost more than 90% of its value, while food prices have increased by more than 50%.^(41)^ In fact, 42.97% of the nurses in our sample reported an insufficient monthly income. Therefore, it is likely that the double crisis significantly affected the emotional well-being of the Lebanese nurses and eventually increased their anxiety, depression and stress levels even beyond that of the general Lebanese population (anxiety and depression of 42% and 42.6%, respectively).^(2)^

The main aim of our study was to investigate whether EE was associated with mental health. Indeed, our study revealed that EE was associated with depression which is consistent with the existing body of literature that has highlighted the association between depressive symptoms and EE.^(44,45)^ Furthermore, depression was correlated with weight gain in our sample of nurses. According to previous studies, this association could be mediated by emotional eating.^(31)^ Additionally, earlier studies have associated depression with unhealthy dietary choices. For example, a previous study highlighted the correlation between depressive symptoms and lower consumption of healthy items such as fruits and vegetables.^(44)^ In addition, two community surveys have demonstrated that greater severity of depressive symptoms was linked to a decreased probability of adhering to dietary recommendations.^(46)^ These results are consistent with our study outcome, where depressed nurses consumed fewer nuts and dishes prepared with sofrito sauce.

Previous studies have shown that women were more stressed about the pandemic and social isolation than males.^(47,48)^ Likewise, our study detected a gender disparity towards anxiety and stress, with female nurses being more affected than their male counterparts. Our results are consistent with previously reported findings among Iranian nurses and medical staff in China during the COVID-19 outbreak where females were more stressed, anxious and depressed than males.^(39)^ Based on functional magnetic resonance imaging to study brain responses, men and women may be equally susceptible to stress and anxiety,^(49,50)^ although this susceptibility may be revealed through distinct mental conditions. Accordingly, women are more likely than males to have stress-related psychological conditions, such as stress and anxiety disorders, throughout their lifespan,^(51)^ whereas men are more likely to experience behaviours that are externalised (such as substance misuse and aggression.^(52)^

### Strengths and limitations

The current study is the first to our knowledge to assess EE and mental health and their correlating factors in relation to nurses working in Lebanese hospitals during the double crisis. However, some limitations should be noted. First, the nurses took part voluntarily in this study, and selection bias therefore cannot be overlooked. Second, data was self-reported in order to explore emotional eating and mental states among the participants. Similarly, nurses reported their own weight and height. Finally, due to the cross-sectional design, causal relationships cannot be clearly elucidated, therefore longitudinal studies are warranted toward an in-depth investigation of the relationship between different variables studied.

## Conclusion and recommendations

In conclusion, this study revealed that females, non-smokers and depressed nurses had a higher tendency towards EE. Additionally, it was found that EE is associated with weight gain and increased food intake, specifically the consumption of fried foods. With regard to mental health, gender disparity was present, with females experiencing more stress and anxiety than their male counterparts. Furthermore, depression was associated with weight gain and lower consumption of healthy food. Finally, EE appeared only to be correlated with depression. Interventional programmes are thus warranted in order to improve eating habits while reducing psychological discomfort among nurses, especially during crisis. Healthcare systems should offer nutrition education and nutritional counselling if needed and provide healthy snacks and meals at workplace in order to lessen the emotional eating burden experienced during stressful situations. In addition, these programmes could focus on self-management techniques to teach nurses, among other healthcare workers, to self-regulate their eating habits. Self-regulation programmes have been successful in improving physical activity levels and sustaining weight reduction, both of which are associated with mood enhancement.^(53)^ Psychological counselling, stress management sessions and emotional intelligence courses should also be provided to help nurses cope with negative feelings that arise during emergency situations. All these strategies can reduce stress, anxiety and depression levels which will improve the performance of nurses and ultimately lead to improvement in the quality of the healthcare delivered. In fact, previous studies highlighted the importance of emotional intelligence for adequate cognitive performance. In this regard, an Iranian study has highlighted the importance of the ability to control one’s emotions for academic achievement.^(54)^ Furthermore, to help nurses face future emergencies without compromising their mental well-being, it is important to empower them with disaster competencies. In fact, nurses may require training in this specific area. For example, a study conducted in Iran revealed that nursing students have poor disaster competencies.^(55)^ Therefore, the curriculum of the nursing programme should be revised to encompass well designed practical courses tackling disaster competencies and emotional intelligence during critical situations. In order to guide the Lebanese authorities to develop well-tailored preparedness plans for future unexpected emergency situations, future studies should focus on gaining an in-depth view of the problem and its nature in the Lebanese context as well as assessing the impact of various intervention programmes on the nurse’s well-being.

## Abbreviations

**EE:** Emotional Eating; **MD:** Mediterranean diet; **NCD:** non-communicable diseases; **EES:** Emotional Eating Scale; **TS:** Technique Superieur; **BT:** Baccalaureat Technique; **LT:** License Technique; **BS:** Bachelor of Science; **PHQ-9:** Patient Health Questionnaire-9; **GAD-7:** Generalised anxiety disorder-7; **PSS-10:** Perceived Stress Scale-10; **BMI:** Body Mass Index; **WHO:** World Health Organization.
